# Perivascular Adipose Tissue Oxidative Stress in Obesity

**DOI:** 10.3390/antiox12081595

**Published:** 2023-08-10

**Authors:** Andy W. C. Man, Yawen Zhou, Ning Xia, Huige Li

**Affiliations:** Department of Pharmacology, Johannes Gutenberg University Medical Center, 55131 Mainz, Germany; wingcman@uni-mainz.de (A.W.C.M.); yawezhou@uni-mainz.de (Y.Z.); xianing@uni-mainz.de (N.X.)

**Keywords:** antioxidant, reactive oxygen species, eNOS, browning, redox balance

## Abstract

Perivascular adipose tissue (PVAT) adheres to most systemic blood vessels in the body. Healthy PVAT exerts anticontractile effects on blood vessels and further protects against cardiovascular and metabolic diseases. Healthy PVAT regulates vascular homeostasis via secreting an array of adipokine, hormones, and growth factors. Normally, homeostatic reactive oxygen species (ROS) in PVAT act as secondary messengers in various signalling pathways and contribute to vascular tone regulation. Excessive ROS are eliminated by the antioxidant defence system in PVAT. Oxidative stress occurs when the production of ROS exceeds the endogenous antioxidant defence, leading to a redox imbalance. Oxidative stress is a pivotal pathophysiological process in cardiovascular and metabolic complications. In obesity, PVAT becomes dysfunctional and exerts detrimental effects on the blood vessels. Therefore, redox balance in PVAT emerges as a potential pathophysiological mechanism underlying obesity-induced cardiovascular diseases. In this review, we summarise new findings describing different ROS, the major sources of ROS and antioxidant defence in PVAT, as well as potential pharmacological intervention of PVAT oxidative stress in obesity.

## 1. Introduction

Obesity is now known as an epidemic worldwide, which has become a global public health concern and burden [1]. Obesity is a well-known risk factor for cardiovascular disorders like endothelial dysfunction, atherosclerosis, hypertension, and coronary artery disease [2]. In 1991, the pioneering work of Soltis and Cassis suggested that perivascular adipose tissue (PVAT), a functional specialised ectopic fat depot, acts as a critical modulator of vascular physiology and pathology [3]. Indeed, accumulating data from both clinical and experimental studies demonstrate that the dysfunction of PVAT is a causal link between metabolic diseases and cardiovascular complications [4,5,6]. Most blood vessels, including large arteries and veins, small and resistance vessels, and skeletal muscle microvessels, are surrounded by PVAT [7]. PVAT stays in close proximity to the tunica adventitia of blood vessels, serving as a pivotal endocrine and/or paracrine tissue that maintains cardiovascular and metabolic homeostasis. PVAT contains both white and brown adipocytes [8]. Apart from adipocytes, endothelial cells, fibroblasts, immune cells extracellular matrix, and adrenergic nerves endings are also present in PVAT. Depending on the vessel type and region, PVAT may have different compositional, phenotypic, and functional aspects throughout the vascular system [9,10]. The phenotype of PVAT has been extensively reviewed [11,12,13,14,15]. In recent decades, revealing the crosstalk between blood vessels and PVAT has become a particular interest in the field of vascular biology. Apart from its structural and mechanical roles in vascular support, PVAT is actively involved in vascular homeostasis and contributes to vascular dysfunction associated with cardiovascular and metabolic diseases.

A healthy PVAT Is known to exert anticontractile effects on blood vessels in both animal models and humans [16,17]. PVAT, as a endocrine and/or paracrine tissue, regulates vascular function by releasing various vaso-active factors, including adipokines, chemokines, cytokines, hydrogen sulphide (H_2_S), nitric oxide (NO), and reactive oxygen species (ROS) [7]. These vasoactive substances could enter the endothelial layer of the vessel wall by diffusion or via the vasa vasorum or the small media conduit networks that connect the media with the adventitia layer [9,18,19]. These factors produced from PVAT include proinflammatory and anti-inflammatory molecules, which take part in various cellular processes, including smooth muscle proliferation and migration, vascular tone, inflammation, and oxidative stress in the vasculature [10,20].

Depending on the ‘health status’ of PVAT, it may elicit beneficial or harmful effects on the vasculature [21]. In obesity, PVAT becomes dysfunctional and exerts detrimental effects on the blood vessels [4,5,6]. The ‘obesity triad’ is proposed as the central mechanism in obesity-induced PVAT dysfunction [7]. The obesity triad consists of the interactions among PVAT hypoxia, inflammation, and oxidative stress. Among the triad, oxidative stress is a pivotal pathophysiological process in cardiovascular and metabolic complications, including obesity, type 2 diabetes, and hypertension. As oxidative stress is a key feature of hypertension, it is also known to regulate redox-dependent inflammatory molecules [22]. Normally, homeostatic ROS act as crucial secondary messengers in different signalling pathways of both innate and adaptive immune responses [23]. ROS can be generated from the mitochondria, nicotinamide adenine dinucleotide phosphate oxidase (NOX) system, and endothelial nitic oxide synthase (eNOS) uncoupling in PVAT [24]. Oxidative stress occurs when the production of ROS exceeds the capacity of the endogenous antioxidant defence, leading to a redox imbalance [24]. Oxidative stress can attenuate the anti-contractile effect of PVAT [17]. Therefore, targeting oxidative stress in PVAT could be a therapeutic strategy for preventing obesity-related cardiovascular diseases in the future. In this review, we summarise recent findings (mainly based on a PubMed database search of the literature from 2013 to 2023 using the keywords ‘PVAT’ and ‘oxidative stress’) on ROS-generating systems and antioxidant defence in PVAT and oxidative stress in PVAT during obesity, and we discuss the potential pharmacological treatments by targeting PVAT in cardiovascular and metabolic diseases.

## 2. ROS-Generating Systems in PVAT

By far, the main described sources of ROS in PVAT are mitochondria, the NOX family of NADPH oxidase, and eNOS uncoupling. Mitochondrial ROS in adipose tissues have been well described, especially in brown and beige adipocytes [25], while adipocytes in PVAT also generate mitochondrial ROS. Mitochondria are known as crucial intracellular regulators of energy metabolism [26], and have emerged as organelles that play critical roles in cellular responses to different stimuli [27]. It is known that adenosine triphosphate (ATP) production in mitochondria can oxidise substrate by oxidative phosphorylation in the electron transport chain (ETC) [28]. The ETC generates a proton motive force by pumping protons from the matrix to the intermembrane space by oxidative phosphorylation. Mitochondrial proton and electron leak may have major effects on mitochondrial coupling efficiency and ROS production. Protons may re-enter the matrix without going through the ATP synthase and losing ATP production [29]. Unpaired electrons can react with oxygen to form ROS (mainly superoxide O_2_^−^) [29]. Superoxide can then be dismutated to hydrogen peroxide (H_2_O_2_) and generate hydroxide (OH^−^) and hydroxyl (OH^•^) radical by Fenton reaction [29]. Less than a decade ago, Costa et al. first demonstrated the role of PVAT mitochondria as a source of ROS [30]. Mitochondrial ROS have been implicated in the regulation of vascular tone (vasoconstriction and vasodilation) [30,31,32,33], cell growth, and migration [34]. By using oxidative phosphorylation uncouplers, the authors have demonstrated that mitochondrial-derived ROS in thoracic PVAT can, at least partly, modulate the contractility of vascular smooth muscles [30]. Mitochondrial uncoupling protein 1 (UCP-1) is the hallmark of brown adipocytes and is responsible for cold- and diet-induced thermogenesis. In thoracic PVAT, the gene expression pattern is almost identical to brown adipose tissue (BAT) in mice, and PVAT from human coronary artery also expresses UCP-1 [35]. A recent study showed that a deficiency of UCP-1 led to an overproduction of mitochondrial ROS and exacerbated obesity-related vascular dysfunction and atherosclerosis [36]. In addition, in a mice model of interleukin (IL)-18 knockout, ROS production in the PVAT was augmented and accompanied by the deformation of PVAT mitochondria and PVAT whitening [37]. These findings suggest that mitochondrial ROS may play important roles in both vascular and PVAT homeostasis, while modulating mitochondrial biogenesis may be critical in maintaining normal PVAT function.

**Figure 1:**
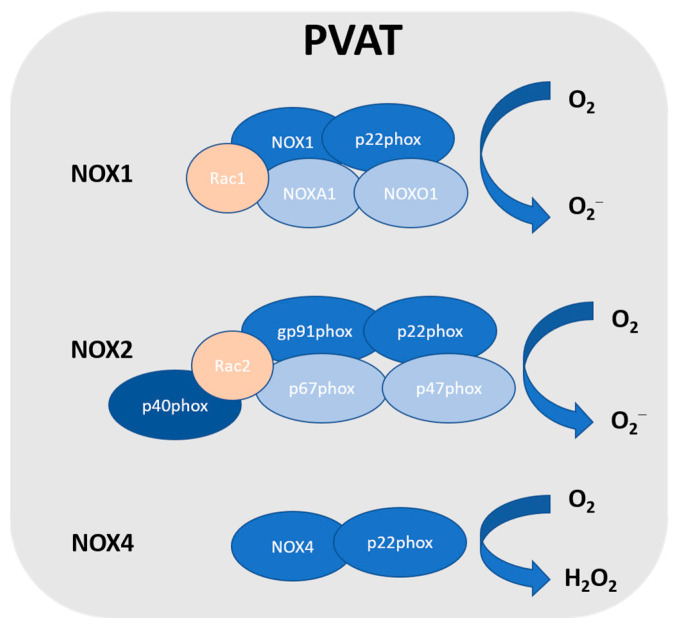
NADPH oxidase in PVAT. So far, NOX1, NOX2, and NOX4 have been detected in PVAT. NOX2 is the prototype NADPH oxidase, and it is a complex that comprises several subunits, including Rac, p47phox, p40phox, p67phox, p22phox, and the catalytic subunit gp91phox. NOX1 is the closest NOX2 homologue, which includes the catalytic subunit NOX1, subunit p22phox, Rac1, NADPH oxidase activator 1 (NOXA1), and NADPH oxidase organiser 1 (NOXO1). NOXA1 is structurally homologous to p67phox, while NOXO1 is structurally homologous to p47phox. NOX4 is a constitutively activated isoform that consists of catalytic subunit NOX4 and subunit p22phox. Unlike other NOX, due to the rapid conversion, NOX4 generates hydrogen peroxide.

In vasculature, the NOX family is one of the major sources of ROS [38]. NOX utilises NADPH as an electron donor to catalyse the production of O_2_^−^ [39,40,41]. NOX2 is the prototype NADPH oxidase, and it is a complex that comprises several subunits, including Rac, p47phox, p40phox, p67phox, p22phox, and the catalytic subunit gp91phox [42]. Upon stimulation, p47phox is phosphorylated, which triggers the complex formation of the cytosolic subunits (p47phox, p40phox and p67phox) followed by translocation to the membrane. This complex is then associated with gp91phox and p22phox to generate superoxide. NOX1 is the first described and the closest NOX2 homologue [43]. Currently, there are seven homologues of NOX identified in humans: NOX1-5 and dual oxidase (Duox1 and 2) [44,45]. NOX4 releases H_2_O_2_, while other NOX isoforms generate superoxide [46]. These nonphagocytic NOXs produce superoxide constitutively and intracellularly [47]. NOX1, NOX2, NOX4, and NOX5 are expressed in vascular cells [38], while only NOX1, NOX2, and NOX4 are detected in PVAT [48] (Figure 1). So far, NOX5 has not been detected in rodents, and there have been no reports on NOX5 in human PVAT. However, the detailed role of NOX in PVAT is not well known. NOX-derived ROS in PVAT was first reported by Gao et al. [49]. An inhibitor of NOX exerted greater inhibition on electrical field stimulation (EFS)-induced contractions in PVAT-attached mesenteric arteries, which was associated with the attenuation of EFS-induced superoxide generation from the PVAT [49]. The authors also demonstrated that p67phox was localised in the cytoplasm and cell membrane of adipocytes from mesenteric PVAT [49]. NOX-derived ROS in PVAT have been shown to induce endothelial dysfunction by scavenging NO released from the endothelium and modulating perivascular inflammation [50]. In a mice model of p22phox subunit overexpression, it was shown that the augmented hypertension was associated with enhanced vascular ROS production and increased PVAT leukocyte infiltration [51]. On the other hand, a mice model with NOX deficiency (such as p47phox subunit, NOX1, and NOX4) showed beneficial effects against hypertension [52,53]. Surprisingly, a recent study reported that the inhibition of NOX1/4 led to an increase in blood pressure associated with PVAT inflammation and accelerated vascular aging in rats, which was also associated with the upregulation of the expressions of proinflammatory chemokines (C-C motif chemokine ligands CCL2 and CCL5) in the PVAT [54].

**Figure 2:**
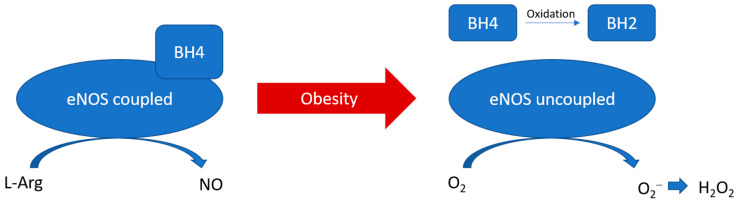
PVAT eNOS uncoupling during obesity. During obesity, BH4 is oxidised to BH2. The reduced availability of arginine and BH4 leads to the uncoupling of eNOS, which switches to produce superoxide instead of NO.

eNOS is a homodimer, heme-containing oxidoreductase that catalyses the conversion of L-arginine and O_2_ to L-citrulline and NO [55]. NO is a crucial modulator in vascular homeostasis, which is known to inhibit vascular smooth muscle proliferation and migration, leukocyte adhesion, platelet aggregation, and inflammation [56]. The oxygenase domain of eNOS binds with L-arginine and the cofactor tetrahydrobiopterin (BH4), while the reductase domain possesses sites for the electron donors NADPH, flavin adenine dinucleotide (FAD), and flavin mononucleotide (FMN) [55]. The oxygenase and reductase domains are connected by a calcium-complexed calmodulin binding site, while calcium-activated calmodulin facilitates the interdomain electron transfer and NO synthesis. This reaction is referred to as eNOS coupling in normal conditions [57]. The stabilisation of the eNOS dimer is essential for eNOS coupling. The coupling of eNOS is dependent on the protein–protein interaction and the availability of arginine and BH4. On the other hand, uncoupled eNOS refers to the situation that the flavins electron transfer is uncoupled to L-arginine oxidation, switching to superoxide production instead of NO [57]. Superoxide can then be quickly converted to H_2_O_2_ by superoxide dismutase (Figure 2). Indeed, eNOS is expressed, but not exclusively, in vascular endothelial cells. Recently, eNOS expression has been reported in cells other than endothelial cells in vitro and in vivo. In particular, eNOS expression and NO production have been detected in the adipocytes in aortic PVAT in both animal models and human samples [58,59,60,61,62]. The expression of eNOS in PVAT highly varies among the anatomical localisations in the vascular system. Abdominal aortic PVAT seems to have a lower eNOS expression compared to that of thoracic aortic PVAT, whereas the expression of eNOS remains the same along the vessel wall itself [63]. In addition, unpublished data from our group suggest a comparable level of eNOS expression between mesenteric PVAT and thoracic aortic PVAT.

## 3. Type of ROS in PVAT

Currently, there are different methods that have been used in studies to detect and measure ROS levels. The most commonly used methods to detect ROS in PVAT and other tissues include chemiluminescent assays (e.g., 5-amino-2,3-dihydroxy-1,4-phthalayineidone (luminol) is used to detect O_2_^−^), fluorescent probes (e.g., dihydroethidium (DHE) and MitoSOX are used to detect O_2_^−^; Amplex red is used to detect H_2_O_2_; DCF-DA is used to detect OH^•^), and electron paramagnetic resonance spin trapping (EPR is used to detect O_2_^−^ or OH^•^) spectroscopy [64,65,66,67,68]. Each of these methods has their its specificities for different ROS and limitations.

As mentioned above, significant production of O_2_^−^ and H_2_O_2_ within the PVAT has been detected in various studies. H_2_O_2_ can be converted into hydroxide (OH^−^) and hydroxyl (OH^•^) radical by Fenton reaction, which can oxidase DNA and lipids and cause cell damage [29,69]. In addition, O_2_^−^ can react with NO to generate a highly reactive nitrogen species peroxynitrite (ONOO^−^), which is also a strong oxidant [70]. ROS production and lipid peroxidation levels appear to be similar in the PVAT along the aorta [63], while it may be different among the PVAT of different vascular beds. Under normal conditions, O_2_^−^ favours vasoconstriction, while H_2_O_2_ contributes to vasodilatation via the activation of the potassium channel and is considered an endothelium-derived hyperpolarising factor (EDHF) [71]. Also, mitochondrial ROS can act as inter- and intracellular signals in vital cellular processes, partly by oxidising redox-sensitive protein phosphatases and kinases, which in turn modulate the phosphorylation of transcription factors or receptors. Depending on their cellular levels, ROS can be either beneficial or deleterious [27,72]. In the presence of cardiovascular risk factors and in pathological conditions, the redox balance in PVAT is disturbed, leading to ROS overproduction and causing oxidative damage to the PVAT itself and the adjacent vessel walls. Indeed, most studies have only measured ROS production in whole PVAT. It would be very helpful for future studies to investigate which cell types in PVAT are responsible for ROS production in healthy conditions and under pathological conditions.

## 4. Antioxidant Systems in PVAT

**Figure 3:**
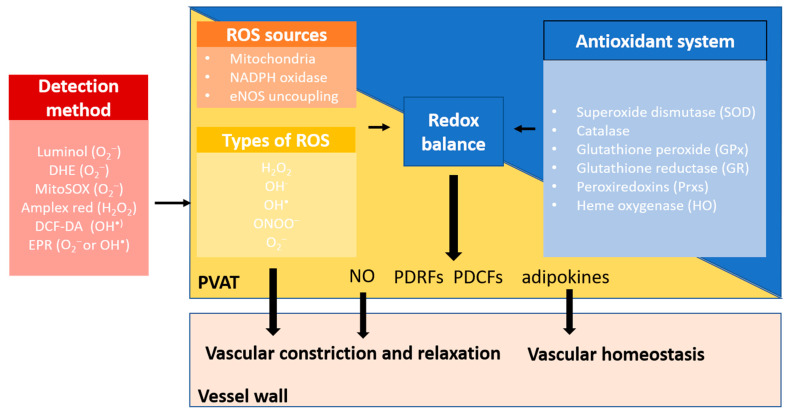
Redox balance in PVAT. PVAT generates different types of ROS through multiple sources, including mitochondria, NOX family of NADPH oxidase, and eNOS uncoupling. Physiological levels of ROS are required for normal PVAT function. Excessive ROS are eliminated by the antioxidant defence system in PVAT. These endogenous antioxidant enzymes, including superoxide dismutase (SOD), catalase, glutathione peroxide (GPx), glutathione reductase (GR), peroxiredoxins (Prxs), and heme oxygenase (HO), are important antioxidant defences that reduce the intracellular ROS burden. Under normal conditions, O_2_^−^ favours vasoconstriction, while H_2_O_2_ contributes to vasodilatation as an endothelium-derived hyperpolarising factor (EDHF). PVAT also releases nitric oxide (NO), PVAT-derived contracting factors (PDCFs), and relaxing factors (PDRFs) and adipokines that are responsible for modulating vascular tone and regulating vascular homeostasis. Healthy PVAT with a balanced redox status is crucial to maintain the normal function of PVAT.

The maintenance of the ROS level is achieved by the endogenous antioxidant enzymes, including superoxide dismutase (SOD), catalase, glutathione peroxide (GPx), glutathione reductase (GR) peroxiredoxins (Prxs), and heme oxygenase (HO). These enzymes are important antioxidant defences that can reduce the intracellular ROS burden [73]. Currently, the antioxidant system in PVAT has received less attention in the studies of cardiovascular and metabolic diseases (Figure 3).

SOD is a superoxide-scavenging enzyme that catalyses the dismutation of O_2_^−^ into molecular oxygen and H_2_O_2_. PVAT expresses CuZn-SOD (SOD1), Mn-SOD (SOD2), and EC-SOD (SOD3) [49,74]. In mice with interleukin (IL)-18 deficiency, the anti-contractile function of PVAT was impaired in association with decreased SOD2 expression in deformed mitochondria in PVAT and increased PVAT whitening [37]. However, mice with adipocyte-specific SOD2 deficiency exhibited resistance to HFD-induced obesity and enhanced energy expenditure [75]. This anti-obesity effect of SOD2 deletion in adipocytes was attributed to the activation of mitochondrial biogenesis and the promotion of mitochondrial fatty acid oxidation [75].

Catalase, mainly present in peroxisomes, eliminates excessive H_2_O_2_. Catalase expression in PVAT was reported [30,76]. In a study, the authors detected a decreased expression of catalase in norephinephrine (NE)-stimulated PVAT, which was associated with increased H_2_O_2_ (detected by Amplex Red) [30]. Extracellular catalase treatment has been shown to reduce such H_2_O_2_ levels in PVAT [30]. Catalase-knockout mice exhibited increased weight gain and higher fat mass under either normal chow (NCD) or high-fat diet (HFD) feeding than the control [77] and exhibited a prediabetic phenotype [78]. Unfortunately, these studies only reported the phenotype of white adipose tissue, without further investigating the function of PVAT.

The GPx family reduces lipid hydroperoxides to alcohols and reduces free H_2_O_2_ to water in a glutathione (GSH)-dependent reduction reaction, while GR acts as a scavenger for OH^•^. The expressions of GPx and GR have been reported in PVAT [79,80]. In mice, the inhibition of GPx resulted in impaired insulin signalling and led to an accumulation of GSH [79]. However, controversial results have been demonstrated in mice models of GPx knockout. Mice lacking GPx-1 have been shown to be protected from high-fat diet-induced insulin resistance [81]. HFD-induced glucose intolerance was improved in mice model with both GPx-1 and catalase deficiency, which was associated with attenuated inflammation and enhanced browning in visceral adipose tissues [82].

Prxs is a ubiquitous family of peroxidase enzyme that modulates the peroxide levels within cells. The catalytic efficiency of Prxs is less than that of catalase [83], but the downregulation of Prxs may lead to a decrease in the rate of H_2_O_2_ catabolism [83]. Among the six members of the Prxs family, Prx1 expression has been reported in human PVAT [84], while the expressions of other Prxs remains to be elucidated. Indeed, Prx2 expression is upregulated during adipocyte differentiation, while the downregulation of Prx2 in adipocytes increased ROS production and inhibited adipogenesis in vitro [85]. Prx3 is localised in the mitochondria and is downregulated significantly in the adipose tissues of obese mice and humans [86]. Prx3 knockout mice showed adipocyte hypertrophy and increased mitochondrial protein carbonylation in vivo, while Prx3 knockdown decreased mitochondrial potential and downregulated adiponectin expression in adipocyte in vitro [86]. Therefore, the expressions and activities of Prx2 and Prx3 in PVAT could be further investigated.

HO is an enzyme catalysing the oxidative degradation of heme to produce free iron, carbon monoxide, and biliverdin. HO-1 is a stress-induced isoform, while HO-2 is a constitutive isoform. HO-1 expression in PVAT has been reported [87,88], while the expression of HO-2 remains unclear. HO-1 overexpression, specifically in adipocyte, attenuated HFD-induce obesity and vascular dysfunction [89]. On the other hand, adipocyte-specific HO-1 knockout exacerbated fasting hyperglycaemia and insulinemia in female mice [90]. Yet, the direct effect of HO-1 manipulation in PVAT requires further investigation.

## 5. PVAT Oxidative Stress in Obesity

Obesity is a condition of excessive fat mass and subclinical inflammation. The prevalence of obesity has doubled worldwide over the past few decades, as well as the concomitant increase in obesity-associated cardiovascular diseases [91]. Obesity is a major risk factor for cardiovascular and metabolic diseases, including type 2 diabetes, insulin resistance, and hypertension [92]. In fact, endothelial dysfunction is not always evident in obese patients in vitro, although they have a higher risk of developing hypertension, cardiomyopathy, and stroke. Various studies have demonstrated that the anti-contractile effects of PVAT are attenuated in obesity [4,58,76]. Indeed, PVAT dysfunction, but not obesity itself, plays an important role in obesity-induced vascular disorders. In mice aortas, the responses to vasodilators were not different between the aortas isolated from obese and lean mice, while vasodilator responses were attenuated in the aortas isolated from obese mice when PVAT was attached [58,62]. In addition, mesenteric arteries incubated with thoracic PVAT from HFD-fed rats showed diminished endothelium-dependent relaxation compared to those incubated with thoracic PVAT from NCD-fed rats [93]. This suggests that the detrimental effects of obesity do not directly influence the intrinsic vascular reactivity but rather the function of PVAT and PVAT dysfunction are closely related to the development of obesity-associated vascular complications.

PVAT function in modulating vascular haemostasis has been extensively reviewed [11,12]. Under normal conditions, the physiological level of ROS is crucial to maintaining vascular homeostasis and is responsible for vascular responses, and excess ROS are antagonised by several antioxidant enzyme systems in PVAT, as mentioned above [49,74]. Pro-inflammatory and pro-oxidative states in PVAT significantly altered the anti-contractile effects and functions of PVAT under obese conditions [94]. For example, during obesity, H_2_O_2_ might act as a PVAT-derived contractile factor [76]. PVAT dysfunction leads to the imbalance of PVAT-derived vasoactive factors and affects vascular function [95].

In obesity, the mass of PVAT is increased and adipocytes become hypertrophy, resulting in a shift to white adipose tissue-like characteristics of PVAT, accompanied by deformed mitochondria [93]. Chronic inflammation is evident in obese PVAT, characterised by the infiltration of dendritic cells and macrophage and the upregulation of inflammatory cytokines, including monocyte chemoattractant protein-1 (MCP-1), tumour necrosis factor alpha (TNF-α), IL-6, and the adipokine leptin [96,97]. On the other hand, the expression of adiponectin, an anti-inflammatory adipokine, is reduced in obese PVAT [98]. Inflammation in PVAT also stimulates the generation of O_2_^−^ and H_2_O_2_ by NOX, which promotes the pro-contractile activity of the vessel wall. Also, hypertrophic adipocytes may exhibit insufficient blood perfusion, which leads to local hypoxia in PVAT. The expression of the key modulator of hypoxia, hypoxia-inducible factor alpha (HIF-1α), is increased in the adipose tissues of obese subjects [99]. HIF-1α can stimulate the production of inflammatory mediators, such as TNF-α and IL-6, and suppress the expression of adiponectin from PVAT [100]. In the small mesenteric arteries of healthy Wistar rats, incubation with TNF-α and IL-6 led to the loss of anti-contractile effects of mesenteric PVAT, whereas the induction of hypoxia led to inflammation and dysfunction of mesenteric PVAT [17]. This hypoxia-induced mesenteric PVAT dysfunction was restored by treatment with either IL-6 antibody, TNF-α antibody, or exogenous catalase and SOD in vitro [17]. HFD-induced obese mice with TNF-α receptor knockdown had reduced H_2_O_2_ generation in PVAT and sensitivity to phenylephrine(PE)-induced vasocontraction, suggesting that oxidative stress is crucial to the pro-contractile shift of PVAT [76]. In addition, the combination of inflammation and oxidative stress may create a vicious cycle that further generates genetic and cardio-metabolic factors, leading to atherogenesis [101]. Therefore, oxidative stress in PVAT is a critical link between metabolic diseases and cardiovascular complications.

Various studies have also demonstrated that obesity-induced PVAT dysfunction is associated with increased ROS generation from different sources [4,58,76,97]. ob/ob mice showed low activity of GPX and the upregulation of gamma-glutamylcysteine synthetase (γ-GCS), resulting in high glutathione content in adipose tissues [79]. In a study, the expression of SOD2 was significantly reduced and catalase expression was increased in the PVAT from obese mice. Interestingly, the SOD activity was increased, while there was no change in the catalase activity in PVAT. These data suggest a compensatory mechanism for increased ROS in obese PVAT [76]. The thoracic PVAT of obese mice lost its anti-contractile effect and became dysfunctional, which was associated with increased levels of O_2_^−^ and H_2_O_2_ detect by DHE, Amplex red, and lucigenin [76]. An excess of mitochondria-derived ROS may be contribution by the oxidative stress in thoracic PVAT, as evidenced by a significant reduction in the O_2_ consumption rate and the downregulation of UCP-1 and SOD2 in this tissue [76]. In addition to mitochondrial ROS, eNOS uncoupling also contributes to oxidative stress in thoracic PVAT. In the thoracic PVAT of obese mice, increased arginase activity was detected, which resulted in eNOS uncoupling, while L-arginine supplementation and arginase inhibition reversed the eNOS uncoupling [58]. In patients who underwent bariatric surgery, obese-induced PVAT dysfunction was restored by increased NO production and reduced TNF-α expression [102]. Moreover, thoracic PVAT-conditioned media from obese mice induced H_2_O_2_ production in the aortas isolated from control mice in vitro [96], suggesting that the secretome from obese PVAT could be pro-oxidant. The abdominal aortic PVAT of HFD-fed mice exhibited increased mass, adipocyte hypertrophy, and increased levels of O_2_^−^ and H_2_O_2_ (evaluated by luminol chemiluminescence technique) compared to NCD-fed mice [4]. The abdominal PVAT from HFD-fed mice was dysfunctional and the abdominal aorta had impaired endothelium-dependent vasodilation in the presence of obese abdominal PVAT. NOX has been suggested as a source of ROS in obese abdominal aortic PVAT, which was evidenced by the upregulation of p67phox subunit [4]. In long-term HFD-fed rats, increased expressions of cytochrome c oxidase, GPx, and UCP-1 and a decreased expression of p22phox were detected in the aortic PVAT [103]. In the early stages of obesity, the overproduction of NO could preserve vascular function in mesenteric arteries [62]. However, in long-term HFD-induced obesity, mesenteric PVAT became dysfunctional and prooxidant, which was associated with increased O_2_^−^ production, increased NOX activity, and reduced SOD activity [97]. HFD-fed mice also showed a reduced expression of SOD3 and glutathione levels in mesenteric PVAT [97]. The dysfunction of mesenteric PVAT in long-term HFD-induced obese mice was attenuated by incubation with exogenous sources of SOD and catalase, suggesting the generation of O_2_^−^ and H_2_O_2_ in these dysfunctional mesenteric PVAT [84]. In addition, proteomic analysis of PVAT from gluteal fat biopsy revealed a downregulation of SOD1 and PRX-1 expression in obese individuals [84].

eNOS in PVAT plays an important role in obesity-induced vascular dysfunction [7,11], and we have recently reviewed the detailed function of eNOS in PVAT both physiological and pathological conditions [12]. Various studies using HFD and/or genetically modified rodent models have demonstrated the pathophysiological role of eNOS expressed in PVAT in modulating vascular tone, function, and homeostasis, inflammation, and oxidative stress [58,104,105]. We have previously shown evidence of PVAT eNOS dysfunction and eNOS uncoupling in mice with HFD-induced obesity [58]. At the early phase of HFD feeding, there was adaptive NO overproduction from mesenteric PVAT in C57BL/6J mice [62], while the expression of eNOS was reduced after long-term HFD feeding in the mesenteric PVAT of obese rats [106] and in the thoracic PVAT of obese mice [97]. The basal production of NO was reduced in the small arteries of obese patients compared to non-obese subjects, while this reduction was only evident in PVAT-adhered and not in PVAT-removed arteries [59]. The upregulation of arginase in obese PVAT reduces the bioavailability of L-arginine for NO production and leads to the uncoupling of eNOS [107], which in turn produces O_2_^−^ and increases oxidative stress in PVAT [58].

Macrophages represent the key modulators of oxidative stress and inflammation in PVAT. The upregulation of IL-6 and MCP-1 levels lead to the recruitment of monocytes and macrophage in PVAT and the subsequent pathology of obesity-induced vascular complications [108,109,110]. Also, a reduced adiponectin level in obese PVAT was associated with increased macrophage infiltration [111]. In obese individuals, mineralocorticoid receptors (MR) are activated and their ligand aldosterone is significantly increased [112]. Aldosterone is known to activate NOX [113] and induce eNOS uncoupling [114]. The upregulation of MR increased H_2_O_2_ generation in adipocytes in vitro [115] and a blockade of MR prevented both mitochondrial and PVAT dysfunction in obesity [116]. MR may participate in PVAT dysfunction through the modulation of mitochondrial function [116]. Also, MR activation in PVAT macrophages may play a critical role in the pathogenesis of obesity-induced vascular dysfunction, as demonstrated by the beneficial effects in myeloid MR KO mice [117]. Therefore, MR activation is especially interesting in the context of obesity-related cardiovascular and metabolic diseases.

The aldoketo reductase super-family catalyses the generation of sorbitol in the polyol metabolic pathway of glucose metabolism. Aldose reductase, a member of the super-family, may deplete the antioxidant glutathione system due to the scavenging of NADPH, which in turn increases ROS production [118]. In a rat model of type 2 diabetes, the aortic PVAT exhibited increased levels of markers of oxidative stress, including malonaldehyde and aldose reductase activity, which were associated with reduced antioxidant defence [110].

**Figure 4:**
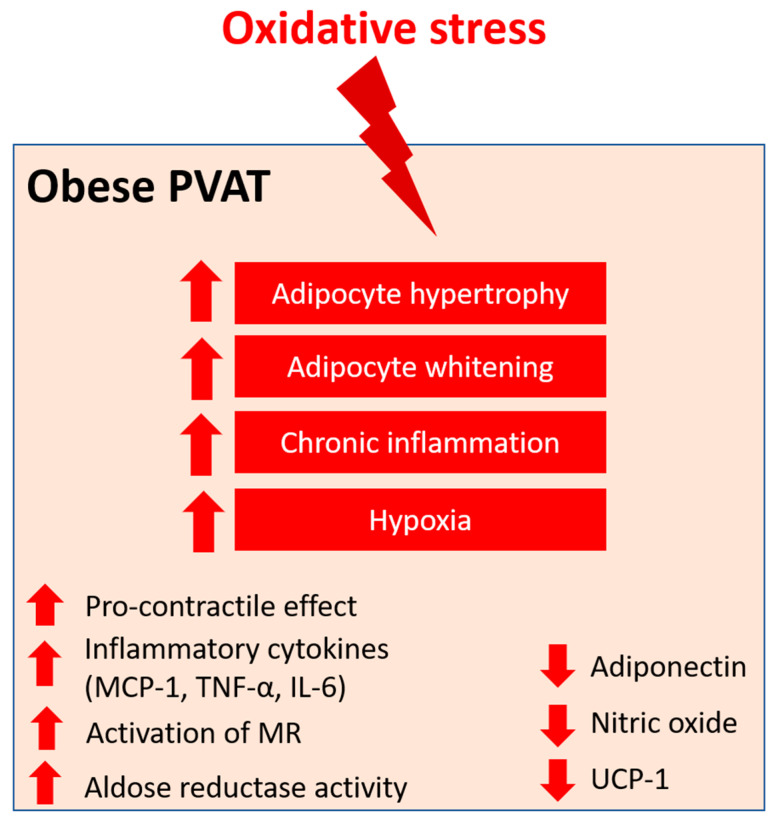
PVAT oxidative stress in obesity. Pro-oxidative state in PVAT significantly alters the anti-contractile effects and functions of PVAT under obese conditions. Obese PVAT becomes hypertrophy and increases whitening of adipocytes. Chronic inflammation and hypoxia are also hallmarks of obese PVAT. In obese PVAT, the anti-contractile effect is lost and becomes pro-contractile. Increased inflammatory cytokines, including MCP-1, TNF-α, and IL-6, are released from PVAT. Also, activation of mineralocorticoid receptors (MR) and increased activity of aldose reductase are recently reported in obese PVAT. The downregulation of UCP-1 in obese PVAT is associated with reduced mitochondrial biogenesis. Obese PVAT also produces less vasoprotective substances like adiponectin and nitric oxide.

Angiotensin II (Ang II) is the key component of the renin–angiotensin–aldosterone system (RAAS), which has been extensively studied in vascular biology. Ang II mediates the PVAT-associated contractile response to perivascular neuronal excitation [119], while adipocyte RAAS is involved in adipogenesis and adipose tissue mass [120]. The upregulation of Ang II during obesity may lead to adipose tissue dysfunction and induce ROS production in PVAT [121]. In a rat model of heart failure, oxidative stress (measured by DHE fluorescence) and reduced NO bioavailability have been shown to be associated with the impaired anti-contractile effect of thoracic PVAT [122]. In a recent RNA sequencing study, the responses of different PVAT to Ang II have been investigated [123]. Upon stimulation by Ang II, abdominal aortic PVAT showed a significant downregulation of mitochondrial genes in oxidative phosphorylation and brown adipocyte markers and an upregulation of inflammatory markers. In addition, Ang II induced even more significant inflammation in both ascending and descending thoracic aortic PVAT [123]. Together, these targets may emerge as possible mediators of oxidative stress in PVAT during obesity, and further studies are warranted to elucidate the mechanisms (Figure 4).

## 6. Pharmacological Prevention of PVAT Oxidative Stress

As discussed above, PVAT dysfunction and the associated vascular complications in obese mice are highly associated with systemic inflammation and oxidative stress in PVAT. Here, we summarise recent studies on potential strategies for targeting PVAT oxidative stress.

### 6.1. Improving Antioxidant Defence

In general, the dismutation of mitochondrial H_2_O_2_, the inactivation of O_2_^−^, and the uncoupling of oxidative phosphorylation have been demonstrated to restore PVAT function and attenuate PE-induced contraction in vessels with PVAT isolated from HFD-fed mice [4,76]. In HFD-induced obese rats, the administration of antioxidative ethanolic extract of Mangosteen pericarp (EEMP), which contains xanthone, has been shown to normalise hypertrophic PVAT and reduce the expression of vascular cell adhesion molecule 1 (VCAM-1) to prevent arterial remodelling [124]. Treatment with an antioxidant, N-acetyl cysteine, normalised the upregulation of angiotensinogen in ROS-treated adipose tissues in both in vitro culture and in vivo obese mice models [125].

Treatment with either enalaprilat (an angiotensin-converting enzyme ACE inhibitor) or candesartan (an Ang II type 1 receptor antagonist) reduced the PVAT-mediated O_2_^−^-induced vasocontraction in rat mesenteric arteries [119]. Also, chronic treatment with quinapril (an ACE inhibitor) reduced the blood pressure and alleviated the potentiation effect of PVAT-mediated superoxide-induced contractions [119]. S-zofenopril, a sulphhydrylated ACE inhibitor, improved vascular function in spontaneous hypertensive rats, which was associated with the potentiation of the H_2_S pathway [126]. The administration of exogenous H_2_S inhibited the generation of ROS and suppressed vascular oxidative stress in hypertensive rats [127]. The antioxidant effect of H_2_S may be attributed to the inhibition of Ang II receptor type 1, the downregulation of NOX, and the upregulation of antioxidant enzymes [128]. Atorvastatin decreases the level of coenzyme Q10, which is a cofactor of H_2_S oxidation, leading to increased H_2_S levels. Atorvastatin treatment has been demonstrated to improve the anti-contractile function of PVAT in spontaneously hypertensive rats [129], while the administration of lipophilic atorvastatin increased H_2_S levels in PVAT and prevented mitochondrial oxidation, which in turn improved the anti-contractile effect of PVAT [130].

Melatonin (5-methoxy-N-acetyltryptamine) is a hormone that has antioxidant activity by promoting direct free radical scavenging and the stimulation of antioxidant enzymes such as SOD [131]. In mice models of accelerated aging, long-term treatment with melatonin normalised the anti-contractile effects of PVAT and was associated with the increased expressions of vasoprotective markers and decreased oxidative stress and inflammation in PVAT [132]. In a recent study, the administration of melatonin restored the anticontractile effect of aortic PVAT in obese rats by reversing the overproduction of ROS, reduced SOD activity, and the decreased bioavailability of NO [133].

Polysaccharide peptides (PsPs) are protein-bound polysaccharide extracted from plants and fungi. The anti-inflammatory, free radical scavenging, and antioxidant properties of PsPs have been demonstrated in different studies [134]. Various studies have shown that PsPs isolated from fungi can restore H_2_O_2_ level by upregulating SOD and catalase expression in the PVAT of HFD-fed rats, which in turn prevents PVAT hypertrophy and arterial remodelling [135,136].

Glucagon-like petide-1 (GLP-1) is a peptide that is mainly produced by the intestinal cells and is known to improve cardiovascular health [137], improve endothelial function in obesity [138], and stimulate fatty acid oxidation and insulin signalling pathways, thus enhancing the antioxidant capacity [139]. An antioxidative GLP-1 analogue, liraglutide, has been demonstrated to attenuate HFD-induced vascular dysfunction by modulating the protein kinase A (PKA)-AMP-activated protein kinase (AMPK)-peroxisome proliferator-activated receptor-gamma coactivator 1alpha (PGC-1α) pathway in obese mice [88]. Liraglutide enhanced the HO-1/adiponectin axis and alleviated HFD-induced oxidative stress in PVAT [88]. Similar findings were reported in another study where liraglutide increased the antioxidant capacity by upregulating the Nrf2/HO-1 pathway in obese mice [88], and alleviated the NLR family pyrin domain containing 3 (NLRP3) inflammasome-dependent inflammation in PVAT by inhibiting nuclear factor (NF)-κB signalling [140]. Exendin-4, another GLP-1 analogue, reduced the expressions of inflammatory and oxidative markers (such as NOX4) in in vitro and in vivo experiments [141]. On the other hand, dipeptidyl peptidase 4 (DPP-4), an enzyme secreted from PVAT, degrades GLP-1 and has been suggested as a pathophysiological link between obesity and cardiovascular diseases [142]. DPP-4 inhibitors have been shown to exert direct antioxidant effects in rodent models [143,144]. The administration of teneligliptin, a DPP-4 inhibitor, attenuated atherosclerosis progression in apolipoprotein E (ApoE) knockout mice by alleviating inflammation and oxidative stress in both the vasculature and PVAT [145]. These studies suggest that enhancing GLP-1 activity and/or downregulating DPP-4 in PVAT may improve PVAT function by alleviating inflammation and oxidative stress.

### 6.2. Restoring eNOS Function

PVAT dysfunction can be rescued by restoring the normal expression and function of eNOS. In mice lacking low-density lipoprotein receptors (Ldlrs), thoracic PVAT exhibited compensatorily increased eNOS expression and NO production, which protected against impaired vasodilatation responses to acetylcholine and insulin [60]. Standardised Crataegus extract WS^®^ 1442 is a dry extract from hawthorn leaves with flowers with antioxidative properties [146]. Our lab has previously demonstrated that WS^®^ 1442 treatment can restore the vascular function in PVAT-attached aorta rings isolated from HFD-induced obese mice, partly by reversing the reduced Akt (protein kinase B) phosphorylation, reduced eNOS phosphorylation, and enhanced eNOS acetylation in PVAT [147].

The plasma levels of adiponectin and adiponectin expression in adipose tissues are significantly diminished in eNOS global knockout mice [148]. Long-term adiponectin treatment in HFD-fed rats normalised NO-dependent vasorelaxation, which was associated with decreased PVAT inflammation and enhanced eNOS phosphorylation [149]. In a recent study, treatment with methotrexate, an anti-inflammatory drug with antioxidant effects, rescued endothelial and PVAT dysfunction and adipokine dysregulation via activating the AMPK/eNOS pathway in PVAT [150]. Also, treatments with various modulators of AMPK activity, including 5-Aminoimidazole-4-carboxamide ribonucleotide (AICAR), diosgenin, metformin, methotrexate, resveratrol, and salicylate, have been shown to increase the anticontractile function of PVAT in different studies [151,152]. In addition, irisin, a newly identified hormone secreted by myocytes, has been shown to attenuate PVAT dysfunction in HFD-induced obese mice via the upregulation of the HO-1/adiponectin axis and browning of the PVAT [87]. In another study, irisin improved endothelial function in obese subjects via activation of the AMPK-eNOS pathway [153], suggesting that the administration of irisin may improve PVAT function by activation of the AMPK-eNOS pathway in PVAT.

Moreover, the expression of eNOS was revealed in both BAT and isolated brown adipocytes [154], whereas eNOS-derived NO has been shown to promote adiponectin synthesis and play a crucial role in mitochondrial biogenesis [155]. These results suggest that restoring eNOS function may also facilitate thermogenesis and browning in PVAT.

### 6.3. Restoring Mitochondrial Function and Browning of PVAT

During obesity, PVAT mainly displays white adipose tissue-like phenotypes, while stimulating the white to brown characteristics or maintaining beiging in PVAT can be a critical strategy to maintain and restore the function of PVAT. In obesity, PVAT resembles white adipose tissue (WAT)-like phenotypes and is associated with augmented oxidative stress and inflammation and reduced NO bioavailability. On the other hand, PVAT with BAT-like phenotypes has been shown to ameliorate oxidative stress and inflammation. Indeed, whitening and browning of PVAT are determined by the mitochondrial function and biogenesis in adipocytes [156].

CDGSH iron-sulphur domain-containing protein 1 (mitoNEET) is a mitochondrial membrane protein that is responsible for regulating the maximal capacity for electron transport and oxidative phosphorylation. In other cell types, mitoNEET has been shown to protect against oxidative stress, possibly by compensating the imbalance in the glutathione system [157]. The expression of mitoNEET is regulated by thermogenic genes such as PGC-1α. The overexpression of mitoNEET in PVAT significantly prevented arterial stiffness and atherosclerosis [158,159]. In mice with mitoNEET overexpression, aortic PVAT exhibited enhanced BAT-like phenotypes, as evidenced by the upregulation of brown adipocyte markers, and counterbalanced Ang II-induced inflammatory and oxidative effects in PVAT [123]. Therefore, potential mitoNEET ligands, including rosiglitazone and resveratrol, may be used to target oxidative stress in PVAT, via restoring mitochondrial function [160].

In addition, cold acclimation is a well-studied stimulus to induce mitochondrial biogenesis and browning in adipose tissues. Cold acclimation has been shown to attenuate HFD-induced endothelial dysfunction and prevent atherosclerosis in mice, which is associated with the downregulation of pro-inflammatory markers in PVAT [161]. Cold acclimation stimulated the browning of the abdominal aortic PVAT in HFD-fed rats by increasing the expressions of UCP-1 and PGC-1α while reducing the expressions of TNF-α, IL-6, and p65 [162]. Also, cold acclimation stimulates glucose uptake and triglyceride clearance in adipose tissues, which may defend against oxidative stress in PVAT [163,164].

Exercise training has been shown to induce a thermogenic response and adipocyte browning in rat PVAT, and it is associated with enhanced eNOS expression and diminished oxidative stress [165,166,167]. Aerobic exercise training upregulated the protein expression of antioxidant enzymes (including SOD1-3) and decreased ROS generation (measured by DHE fluorescence) in PVAT, and there was an associated improvement in endothelium-dependent vasorelaxation [168]. The beneficial effects of exercise training may be attributed to the increased angiogenesis in PVAT, which improves blood flow, reduces hypoxia and macrophage infiltration [169], and improves vascular function [170]. In addition, diet restriction in male obese rats reduced adipokines and cytokines (including leptin, IL-6, MCP-1, and TNF-α), immune cell infiltration, and the gene expressions of p22phox and p47phox in thoracic PVAT [171]. Moreover, sustained weight loss has been shown to restore PVAT function in obese models [84,102,106,171], possibly by improving the redox status [171] and restoring eNOS expression and NO production in PVAT [106]. These suggest that a healthy lifestyle (i.e., regular exercising, diet control, and weight loss) may prevent and restore obese-induced cardiovascular complications via modulating mitochondrial biogenesis, browning, and eNOS function in PVAT.

PGC-1α and peroxisome proliferator-activated receptor gamma (PPARγ) are important therapeutic targets to restore mitochondrial biogenesis and PVAT function by modulating the browning of adipocytes [172]. Important antioxidant enzymes, including Nrf2/HO-1, are regulated through the PPARγ pathway [173]. PPARγ activation by its agonist, pioglitazone, attenuated obesity-induced arterial stiffening and reduced the inflammatory and oxidative status of PVAT in ob/ob mice [174]. In a mice model of obesity and diabetes, treatment with another PPARγ agonist, rosiglitazone, improved insulin sensitivity, increased serum adiponectin levels, and reduced inflammation in adipose tissues [175]. The expressions of inflammatory genes, including TNF-α, MCP-1, and macrophage antigen-1 (CD11b), in white adipose tissues were reduced in response to rosiglitazone [175].

## 7. Conclusions

**Figure 5:**
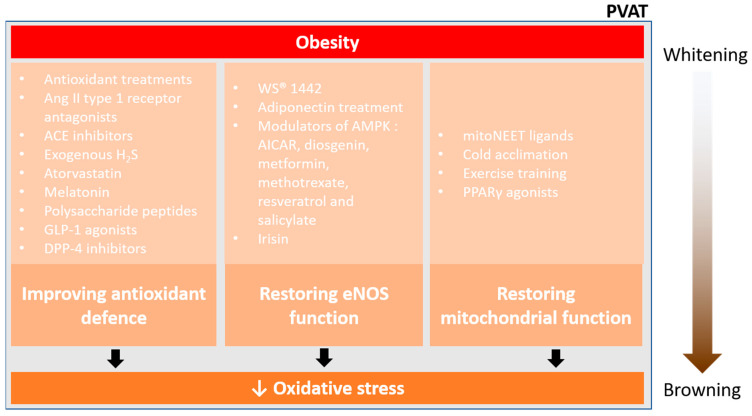
Pharmacological prevention of PVAT oxidative stress. Currently, there are three main ways to reduce oxidative stress and restore PVAT function in obesity, improving antioxidant defence, restoring eNOS function, and restoring mitochondrial function. Obesity enhances the whitening of PVAT, which is associated with inflammation and oxidative stress. Effective pharmacological interventions may reduce the oxidative stress in PVAT, thus facilitating the mitochondrial biogenesis and browning of PVAT.

PVAT has a unique role in modulating vascular homeostasis. In cardiovascular and metabolic diseases, adipose tissues (especially PVAT) dysfunction make a notable contribution to the associated vascular dysfunction. Different studies have provided evidence suggesting that PVAT, in addition to the endothelium, plays a crucial role in the pathophysiology of obesity-induced cardiovascular diseases. In this review, we summarised different ROS sources and the antioxidant defence systems in PVAT. Cardiovascular risk factors may alter the redox balance in PVAT, while oxidative stress in PVAT is a crucial pathophysiological mechanism of cardiovascular complications. Buffering ROS generation in the PVAT may hinder the pathogenesis of obesity-related cardiovascular diseases (Figure 5).

The role of PVAT dysfunction in the pathophysiology of obesity-induced cardiovascular diseases represents a new direction for investigation. Conventional in vitro vascular experiments have mainly been focused on PVAT-denuded vessels, while the function of PVAT has not been focused on in in vivo animal studies. More information is needed to discover novel therapeutic targets in PVAT. The generation of a suitable PVAT-specific transgenic animal model would greatly help the progression of the research field. However, the highly heterogenous origins and regional variations of PVAT in different vascular beds are great challenges that need to be overcome for the creation of PVAT-specific transgenic animal models.

## Data Availability

Not applicable.
